# Investigation of the Association between Drinking Water Habits and the Occurrence of Women Breast Cancer

**DOI:** 10.3390/ijerph17207692

**Published:** 2020-10-21

**Authors:** Yael Keren, Racheli Magnezi, Moshe Carmon, Yona Amitai

**Affiliations:** 1 Department of Management, Bar-Ilan University, Ramat-Gan 5290002, Israel; Racheli.Magnezi@biu.ac.il (R.M.); yonaamitai89@gmail.com (Y.A.); 2 Department of Surgery, Shaare Zedek Medical Center, Jerusalem 9103102, Israel; carmonm@gmail.com

**Keywords:** breast cancer, water drinking, water consumption, liquid consumption, prevention, cancer prevention

## Abstract

Risk and protective factors for breast cancer (BC) include lifestyle, diet, reproduction, and others. Increased risk for colon cancer was linked with low water intake. The link between water consumption and BC was scarcely studied. We investigated the association between water and fluid consumption and the occurrence of BC in a retrospective case–control study in the Shaare Zedek Medical Center, Jerusalem, in 206 women aged 25–65 years (106 with newly diagnosed BC, and 100 controls). A food frequency questionnaire (FFQ), consumption of water, foods, and beverages, lifestyle, and other risk and protective factors were recorded. The age of women in both groups was comparable ((M ± SD) 52.7 ± 9.8 and 50.6 ± 11.4 years, respectively (*p* = 0.29)). Women with BC consumed 20.2% less water (M ± SD = 5.28 ± 4.2 and 6.62 ± 4.5 cups/day, respectively, *p* = 0.02) and 14% less total fluids than controls (M ± SD = 2095 ± 937 mL/day and 2431 ± 1087 mL/day, respectively, *p* = 0.018). Multiple stepwise logistic regression showed that the differences remained significant both for daily water consumption (*p* = 0.031, CI = 0.462–0.964) and for total daily liquid intake (*p* = 0.029, CI = 0.938–0.997). Low water and liquids intake as a risk factor for BC may be related to the younger age of our subjects. The effect of age on the potential role of water intake in decreasing BC risk should be investigated.

## 1. Introduction

Cancer is one of the main causes of mortality worldwide [1,2]. In 2008, 8 million deaths were recorded as a result of malignant diseases, and this figure is estimated to reach 11 million by 2030 [3]. Breast cancer is the most commonly occurring cancer among women [4]. The type of breast cancer depends on which cells in the breast transform into cancer. Invasive breast cancers are a heterogeneous group of tumors that show a wide variation with regard to their clinical presentation, behavior, and morphological spectrum. At least 18 different histological breast cancer types (pathological entities) are described by the World Health Organization (WHO) [5]. Invasive ductal carcinoma not otherwise specified (IDC NOS) accounts for a large majority of breast cancer incidences (50–80%). IDC NOS is a diagnosis by default, being defined by the WHO as a tumor that fails to exhibit sufficient morphological characteristics to be classified into one of the histological special types [5]. Invasive lobular carcinoma is the second most frequent breast cancer (5–15%). In this type of breast cancer, the cells spread from the lobules to the adjacent breast tissues that are close by. These invasive cancer cells can also spread to other parts of the body [6]. Although the disease occurs all over the world, its incidence, mortality, and survival rates vary considerably among different parts of the world, which could be due to many factors such as population composition, lifestyle and nutritional habits, genetic factors, and environment [7].

Breast cancer is the second most common cancer in the world and the most common cancer among women [8]. The risk of breast cancer occurrence in women in the United States is 12.4%, or one in eight women [9]. One million six hundred and seventy thousand new cases of breast cancer were identified worldwide in 2012, accounting for 25% of all cancers [8]. Although cancer exists anywhere in the world, its incidence rate is higher in developed countries, and the incidence rate of breast cancer varies greatly with race and ethnicity [9]. The incidence in Middle Africa and East Asia is 27 per 100,000 and in Northern America is 92 per 100,000 [8].

By 2050, the incidence rate of breast cancer is estimated to reach 3.2 million [7]. With increasing population age in developed countries, the incidence rate of breast cancer among older people is increasing [7]. In 2017, approximately 252,710 new cases of invasive breast cancer and 6341 cases of breast cancer in situ were diagnosed in the United States [10]. Nearly 24% of all breast cancer cases occur in the Asia-pacific region, with the highest rates seen in China, Japan, and Indonesia [11,12]. In addition to Japan, the prevalence of breast cancer is increasing among Asian and American women, with Korea accounting for the highest prevalence of breast cancer from 1988 to 2006 in Southeast Asia from 1988 to 2013 [13]. The one-year survival rate of breast cancer in European countries varies from 94.1% in Scotland to 97.1% in Italy [14]. In African women, survival rate is low due to the delay in seeking diagnosis and treatment for breast cancer [15]. The incidence (age-standardized rate per 100,000) of breast cancer in different regions of the world is as follows: 74.1 in more developed regions, 31.3 in less developed regions, 96.0 in Western Europe, 91.6 in Northern America, 89.4 in Northern Europe, 85.5 in Australia/New Zealand, 28.2 in South-Central Asia, and 27.0 in Eastern Asia [16].

Breast cancer was the fifth leading cause of cancer death in 2012 worldwide, with a record of 324,000 deaths in 2012, and in developed countries it was the most common cause of death. Furthermore, breast cancer was the second cause of death in developed countries, second to lung cancer, with 197,000 deaths accounting for 15.4% of all deaths [8]. The mortality rate of breast cancer is estimated to increase during the next decade in many parts of Europe [17]. Although the prevalence of breast cancer is higher in developed countries, in less developed regions there are higher mortality rates [18]. Furthermore, 89% of deaths from breast cancer in the United States in 2017 occurred in women aged 50 years or older.

Among all risk factors, it has been shown that age is the main risk factor for breast cancer [19]. The median age of diagnosis of breast cancer in women in the United States is 61 years [20]. personal lifestyle and nutritional factors may modify the risk for breast cancer. Mcpherson investigated and summarized risk factors for breast cancer [19], and other studies have reported protective factors that may reduce the risk for breast cancer [21,22,23]. Water intake has been shown to be a protective factor for rectal and colorectal cancer [24,25,26]. In contrast, several studies reported conflicting data regarding the risk for bladder cancer associated with drinking water [27,28].

Water intake includes, approximately, 20% contribution of water from solid foods and 80% contribution of water from beverages and drinking water [29,30,31]. It follows that water intake, although mostly driven by thirst, depends on a variety of factors such as eating and drinking habits and preferences or availability of foods and beverages [32,33,34]. Water loss comes mainly from excretion of water in urine, respiratory water, feces, and sweat [35]. Since the contribution of sweat in water loss is higher in a physically active person and in hot weather [36], water loss is affected by physical activity levels and season. Therefore, water loss is highly variable, even in healthy individuals, depending on the lifestyle of the individual and on environmental conditions or geographical location.

On average, American adults consume 1.1 L (1138 mL) of water as a beverage per day. Older adults (≥71 years) consume less water than younger adults. Generally, men and women consume comparable amounts of water as a beverage. Overall, adults consume 644 mL/d of tap water (about 56% of total water consumed as a beverage) and 502 mL/d of bottled water (44%). Among adults aged 20–50 years, 83% of total water is from beverages, including 37% from plain water, and 17% from moisture in foods. For this age group, soda is an important source of dietary water, accounting for 13% of total water. Coffee and alcohol respectively provide 8.5% and 8% of total water. Among adults aged 51–70 years, 82% of total water comes from beverages, including 32% from plain water, and 18% from moisture in foods. For this age group, soda provides 10% of total water, whereas coffee provides 16% and tea another 9%. Alcohol provides 5% of total water. Among adults aged ≥71 years, 76.0% of total water comes from beverages, including 30% from plain water. In addition, 27% of water comes from moisture in foods. For this age group, soda provides 6% of total water, whereas coffee provides 18% and tea another 7%. Alcohol provides only 2% of total water for this age group. Women aged ≤70 years exceed the Adequate Intake (AI) value, whereas women aged ≥71 years have a shortfall of approximately 603 mL/d. Among adults aged 20–50 years, 40.6% of women fail to meet the Institute of Medicine AI value for total water (3700 mL for men and 2700 mL for women). For adults aged 51–70 years, 44.9% of women fail to meet the AI value for total water. For adults aged ≥71 years, 94.7% of men and 82.6% of women fail to meet the AI value for water [37].

The proposed mechanism for the role of water intake in reducing the risk for colon cancer is through decreasing the gastrointestinal transit time resulting in constipation [38,39]. Constipation may result in slowing the evacuation of potential carcinogens. The study by Maruti et al. showed a tendency towards fewer bowel movements in subjects with breast cancer [40]. The potential mechanism for the protective role of water intake for breast cancer may also be explained by the frequency of bowel motility, similar to the case of colon cancer. It has been shown in previous studies that high levels of estrogen are associated with a higher risk for breast cancer. Chronic constipation is described as fewer bowel movements leading to a decrease of the gastrointestinal transit time with less evacuation of the stool. The less evacuation occurs, the less is the excretion of estrogen too, resulting in higher serum estrogen levels causing stimulation to the growth and division of epithelial breast cells, and by that increasing the risk for breast cancer [40,41,42].

Micozzi et al. showed that among 7702 American women, of whom 123 had breast cancer, fewer bowel movements and hard stools were associated with a higher risk for breast cancer [43]. Additionally, cell hydration has been proposed as a primary factor in the mechanism of carcinogenesis. Increased cell hydration causes cancer not only by promoting cell division and oncogene expression, but also by inactivating genes inducing cell differentiation, and by preventing apoptosis. Conversely, factors that reduce cell hydration prevent cancer by inhibiting cell division and oncogene expression, while activating genes inducing cell differentiation, and by promoting apoptosis [44].

Despite the high frequency of breast cancer and the potential benefit of water consumption as a simple, non-invasive means for reducing the risk for breast cancer, the role of water intake has been scarcely studied. Stookey et al. published a pilot study of 44 patients with breast cancer, compared with 55 controls, which showed that water drinking appeared to confer a beneficial effect on breast cancer risk. The relative risk for breast cancer associated with low water drinking was 0.21, and the risk estimate represented a 4.7-fold difference in the odds of water drinking between cases and controls [45]. The study did not report quantities of water and fluid intake. By contrast, a large-scale cohort study by Maruti et al., where 507 women were diagnosed with breast cancer, did not find an association between drinking water and breast cancer [40]. The current study investigated the association between water and total fluid intake and breast cancer.

## 2. Materials and Methods

We conducted a retrospective case-control study in the pre-operative unit of the Shaare Zedek Medical Center, Jerusalem, Israel. This unit care for patients with breast cancer and other related diseases is one of the leading units in Israel. While officially part of the surgical wing, the unit offers a multi-disciplinary approach with experts in oncology, pathology, and more. Over the course of just one day, the women undergo all necessary tests and examinations. This unit’s working profile allowed to interview diagnosed women with breast cancer in-between the medical process during the same day. In addition, interviewing at this time frame assured to include in this research women with new breast cancer diagnoses.

Participants ranged in age from 25 to 65 years. The study group included 106 women with newly diagnosed, histologically confirmed breast cancer, scheduled for surgery. The control group consisted of 100 women without breast cancer, recruited from healthy women who escorted the patients and from women who came to the unit for other procedures unrelated to cancer. One hundred and nineteen patients were asked to participate in the study. Of these, 106 signed an informed consent and completed the interview (89% compliance). For the control group, participation was offered to 286 women matched by age, of whom 100 consented and completed the interview (35% compliance).

Data collection was conducted from January to December 2015 and was carried out by personal interviews conducted by one of the authors (YK). The interview included a food frequency questionnaire (FFQ) [46]. Information on water consumption was collected by a structured questionnaire for food items and beverages rich in water, which was developed by the Israel Ministry of Health and used in a National Health and Nutritional Survey [47]. Additional data were collected on lifestyle habits, including information about known risk and protective factors. Our questionnaire did not include data on the type of breast cancer since all patients were recruited to the study immediately after having been diagnosed, and thus data were not available. The reason for recruiting patients immediately after the diagnosis was to reflect the lifestyle and nutritional habits prior to the disease that were not affected by the change in their habits after being diagnosed. The study was approved by the Institutional Review Board of the Shaare Zedek Medical Center.

### Statistical Analysis

Descriptive statistics were used to characterize important covariates, including age, nationality, education, BMI, tobacco use, alcohol use, first-degree relatives with history of breast or ovarian cancer, personal history of benign cancer, marital status, number of births, age at menarche and at menopause, reproductive age (between menarche and menopause), contraceptive hormone use, hormonal replacement therapy (HRT) use, constipation, and physical activity.

Quantitative variables were compared using the Mann–Whitney test. Categorical variables were compared using the Chi-Square test. Dichotomous variables were defined using the Fisher’s Exact test. Standard deviations were compared using Levene’s Test for Equality of Variances. Equality of means was compared using t tests. Multi-variable analysis was conducted using a multiple stepwise logistic regression model to evaluate the association of water and total fluid consumption with the presence or absence of breast cancer further.

Covariates with statistical significance were added to the model. All statistical tests were two-sided, with significance defined at 0.05. Statistical analyses were conducted using IBM^®^ SPSS^®^ Statistics 2020, New York, NY, USA.

## 3. Results

Data on personal, lifestyle, and demographic characteristics are presented in Table 1.

The study and control groups were comparable in age, nationality, BMI, education, number of births, and age at menarche and menopause. Alcohol consumption and a family history of breast and ovarian cancer were more frequent in the breast cancer group. Smoking rates and lack of physical activity showed a trend toward being higher among the group with breast cancer.

Data on water and liquid consumption and other nutritional variables are presented in Table 2.

**Figure 1:**
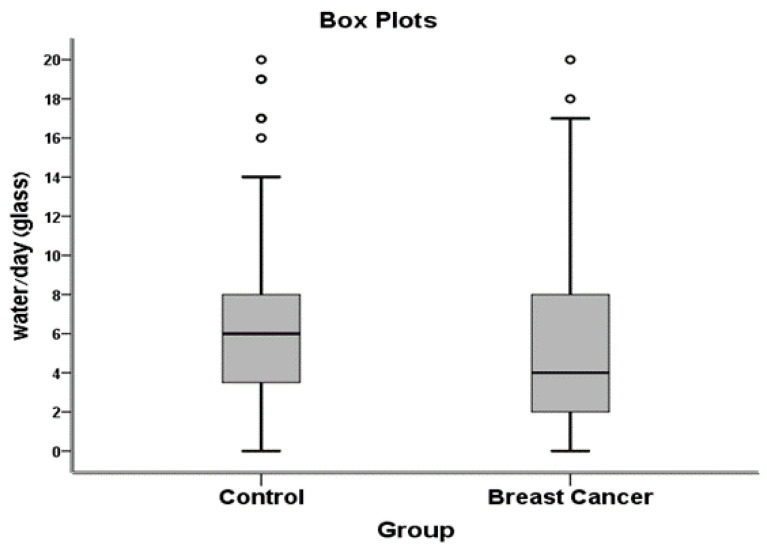
Water consumption (glasses/day).

Significant differences were found between the two groups in water and total fluid consumption. For water consumption, the mean number of cups per day were 5.28 and 6.62 in the breast cancer and control groups, respectively (difference of 20.2%, *p* = 0.02) (Figure 1).

**Figure 2:**
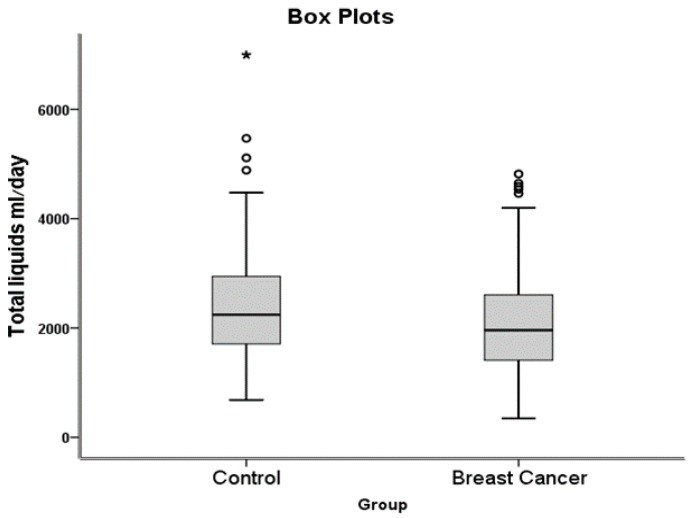
Liquids consumption (mL/day).

Similarly, mean total fluid consumption among subjects with breast cancer was 14% lower than among the control group (2095 vs. 2431 mL per day, respectively, *p* = 0.018) (Figure 2).

Subjects in the breast cancer group consumed fewer fresh fruits than the control group (*p* = 0.003). In a multiple stepwise logistic regression model, the difference in daily water consumption was statistically significant between groups (*p* = 0.031), as was total daily liquid intake (*p* = 0.029). Other variables that remained significant in the logistic regression model were fruit and alcohol consumption and first-degree relatives with breast or ovarian cancer.

## 4. Discussion

Subjects in the breast cancer group reported significantly less water and total fluid intake, compared with the control group. This difference remained statistically significant both for water and for total liquid intake after adjustment for risk factors.

The groups were comparable in most personal and demographic variables (age, BMI, number of births, years of education, ethnic origin, nation, religion). These findings support the observation reported in a small-scale study [45]. In contrast, a large-scale cohort study that included 507 women with breast cancer did not find an association between drinking water and breast cancer [40]. Of note, there were marked differences in the age of the subjects in these studies. In the study by Maruti et al., the mean age of the cohort at baseline when they were cancer free was 61 years, and the age of subjects when breast cancer was diagnosed, sometime during the follow-up period (up to 5 years), was older [14]. In the current study and in that of Stookey et al., patients with breast cancer were a mean age of 52.7 years and 56.5 years at presentation, respectively [45]. Among the breast cancer subjects in our study, 51% were pre-menopausal.

As a rule, the incidence of breast cancer increases with age. In pre-menopausal women, the weight of nutritional risk factors for breast cancer is generally higher than in post-menopausal women [48,49,50]. Thus, the younger age of subjects in our study might explain the observation that low water and fluid consumption may be a risk factor, which may not be the case in older or post-menopausal women.

Several studies found reduced risk for colorectal cancer in association with water consumption [24,25,26]. The data regarding the risk associated with drinking water among individuals with bladder cancer are inconsistent [27,28].

The role of water intake in reducing the risk for breast cancer could be explained by three mechanisms. First, enhancement of gastrointestinal transit time reduces constipation, and it eliminates carcinogens from the gut faster. This mechanism has been proposed as an explanation for the risk reduction for colorectal cancer by increased water intake [38]. Second, cell hydration was suggested as a main factor in the mechanism of carcinogenesis: promoting cell division and oncogene expression, inactivating genes inducing cell differentiation, and preventing apoptosis [44]. Third, frequent bowel motility, leading to decreased gastrointestinal transit time of the gut content, was associated with increased estrogen excretion in the stool and lower serum estrogen levels [40,41,42].

Nutritional risk factors for breast cancer that were previously published include low fruit consumption [51] and high alcohol consumption [52]. These risk factors were also found in our study.

High fruit consumption was found in our study to be associated with a low risk for breast cancer. In contrast, increased risk for breast cancer was found for high alcohol consumption.

While low fruit consumption and high alcohol consumption are recognized as factors for breast cancer, the evidence for low water consumption is scanty. The innovation in our paper is the finding that low water consumption is associated with an increased risk for breast cancer, particularly in younger women.

This study had several limitations. The retrospective design may result in recall bias. However, the study participants in both groups were not aware of the study hypothesis, and the questions regarding water and fluid consumption were embedded among numerous questions on lifestyle and 45 questions on nutritional variables. patients with breast cancer were interviewed within one month of diagnosis, and they were asked to report on their nutritional habits one year earlier, when they were apparently healthy. Data on BRCA were available for only 31 women with breast cancer and 14 in the control group, as this test is not usually offered to women in primary care in Israel. In addition, as women might have been expected to drink more in the summer, answering the questionnaire during the summer months might have resulted in higher reported fluid consumption. However, this might have been counterbalanced with the increased consumption of hot drinks and soups during winter months. To control for possible seasonal drinking differences, the intervention and control groups were interviewed concurrently.

## 5. Conclusions

Our findings that women with breast cancer consumed less water and total fluids compared with controls may suggest a role for water intake in decreasing breast cancer risk. As water consumption was shown to be associated with decreased risk for colorectal cancer, the potential role of water in decreasing the risk for breast cancer is appealing. Drinking water is convenient, harmless, and beneficial on many aspects of health. The finding of low water and liquid intake as a risk factor for breast cancer in our study may be related to the younger age of the participants. The effect of age on the potential role of water intake in the prevention of breast cancer should be investigated further.

## Figures and Tables

**Table 1:** Demographic, personal characteristics, and risk factors of women in the study and control groups.

Parameter		Breast Cancer	Control	Total	*p*-Value
Nationality	Jewish	*N* = 90	*N* = 92	*N* = 182	0.19
	Arab	*N* = 11	*N* = 6	*N* = 17	
	Other	*N* = 5	*N* = 2	*N* = 7	
Age, years (M ± SD)		52.7 ± 9.8	50.6 ± 11.4	51.7 ± 10.6	0.29
		*N* = 106	*N* = 100	*N* = 206	
Education, years (M ± SD)		15.3 ± 3.44	15.4 ± 3.25	15.4 ± 3.34	0.995
		*N* = 106	*N* = 99	*N* = 205	
Number of birth (M ± SD)		3.14 ± 2.42	3.15 ± 2.21	3.15 ± 2.31	0.91
		*N* = 106	*N* = 100	*N* = 206	
BMI (M ± SD)		26.45 ± 6.6	25.92 ± 4.38	26.19 ± 5.63	0.82
		*N* = 106	*N* = 99	*N* = 205	
First degree relative with breast/ovarian cancer	No	62.9%	76.0%	69.3%	0.049
	Yes	37.1%	24.0%	30.7%	
previous benign tumor	No	80.2%	73.0%	76.7%	0.25
	Yes	19.8%	27.0%	23.3%	
Age at menarche (M ± SD)		13.22 ± 1.5	13.14 ± 1.34	13.18 ± 1.44	0.62
		*N* = 104	*N* = 99	*N* = 203	
Age at first pregnancy (M ± SD)		24.92 ± 6.59	24.48 ± 5.85	24.71 ± 6.23	0.14
		*N* = 101	*N* = 94	*N* = 195	
Age at menopause (M ± SD)		50.17 ± 4.88	49.03 ± 4.77	49.57 ± 4.84	0.21
		*N* = 52	*N* = 58	*N* = 110	
Hormone replacement therapy (HRT)	No	*N* = 88	*N* = 84		1.00
	Yes	*N* = 16	*N* = 16		
Contraceptive pills, past/present	No	*N* = 45	*N* = 44		0.89
	Yes	*N* = 60	*N* = 56		
Smoker	present	*N* = 21	*N* = 9	*N* = 30	0.08
	past	*N* = 13	*N* = 12	*N* = 25	
	Never	*N* = 72	*N* = 79	*N* = 151	
Smoking, packs/year (M ± SD)		14.91 ± 13.44	12.57 ± 9.15	14.02 ± 11.94	0.7
		*N* = 34	*N* = 21	*N* = 55	
Alcohol, drinks/week (M ± SD)		0.63 ± 1.4	0.3 ± 0.95	0.47 ± 1.21	0.034
		*N* = 106	*N* = 100	*N* = 206	
Constipation, bowel movements/week (M ± SD)		7.75 ± 4.67	8.07 ± 6.13		0.97
		*N* = 106	*N* = 100		
Physical activity	No	*N* = 46	*N* = 31	*N* = 77	0.06
	Yes	*N* = 60	*N* = 69	*N* = 129	

**Table 2:** Water and total fluid consumption, and other nutritional variables.

Parameter		Breast Cancer	Control	Total	*p*-Value
Water intake, total cups/day	M ± SD	5.28 ± 4.2	6.62 ± 4.5		0.02
		*N* = 106	*N* = 100		
	Median	5.28	6		
Water intake, cups/day	0–3	43	25		0.052
	4–7	34	37		
	≥8	29	38		
Liquid intake (mL/day)	M ± SD	2095 ± 937	2431 ± 1087		0.018
Fiber, g/day		25.84 ± 12.64	25.88 ± 10.38	25.86 ± 11.57	0.7
		*N* = 106	*N* = 100	*N* = 206	
Fish consumption, times/week		1.09 ± 1.48	1.06 ± 1.27	1.08 ± 1.38	0.97
		*N* = 106	*N* = 100	*N* = 206	
Soy, times/week		0.288 ± 0.856	0.21 ± 0.636	0.25 ± 0.757	0.17
		*N* = 106	*N* = 100	*N* = 206	
processed meat, times/week		0.23 ± 0.651	0.27 ± 0.584	0.25 ± 0.618	0.19
		*N* = 106	*N* = 100	*N* = 206	
Fresh fruit, total servings/week		11.17 ± 9.25	14.7 ± 9.7	12.88 ± 9.61	0.003
Dried fruit, servings/week		1.81 ± 3.29	2.16 ± 3.3	1.98 ± 3.29	0.5
Fruit, servings/week	0–6	31.10%	14%	22.80%	0.006
	22–49	10.40%	20%	15%	
	7–21	58.50%	66%	62.10%	
Vegetables, times/week	19.8 ± 11.07		17.3 ± 9.51	18.54 ± 10.34	0.07
Milk consumption, cups/week	4.96 ± 5.64		5.28 ± 3.85	5.12 ± 4.8	0.110
	*N* = 106		*N* = 100	*N* = 206	
Dairy products, servings/week		3.73 ± 2.46	4.17 ± 3.62	3.94 ± 3.08	0.87
		*N* = 106	*N* = 100	*N* = 206	

## References

[1] Momenimovahed Z., Salehiniya H. (2017). Incidence, mortality and risk factors of cervical cancer in the world. BioMed Res. Ther..

[2] Momenimovahed Z., Ghoncheh M., Pakzad R., Hasanpour H., Salehiniya H. (2017). Incidence and mortality of uterine cancer and relationship with Human Development Index in the world. Çukurova Med. J..

[3] Benson J.R., Jatoi I. (2012). The global breast cancer burden. Future Oncol..

[4] WHO: Breast Cancer: Prevention and Control. http://www.who.int/cancer/detection/breastcancer/en..

[5] Tavassoli F.A., Devilee P. (2003). World Health Organization Classification of Tumors. Pathology and Genetics of Tumors of the Breast and Female Genital Organs.

[6] Schnitt S.J., Guidi A.J., Harris J.R., Lippman M.E., Morrow M., Osborne C.K. (2004). Pathology of invasive breast cancer. Diseases of the Breast.

[7] Hortobagyi G.N., de la Garza Salazar J., Pritchard K., Amadori D., Haidinger R., Hudis C.A., Khaled H., Liu M.C., Martin M., Namer M. (2005). The global breast cancer burden: Variations in epidemiology and survival. Clin. Breast Cancer.

[8] Ferley J., SoerjomataramI I., Ervik M., Dikshit R., Eser S. (2013). GLOBOCAN 2012 v1.0. Cancer Incidence and Mortality Worldwide: IARC Cancer Base No. 11.

[9] DeSantis C., Ma J., Bryan L., Jemal A. (2014). Breast cancer statistics, 2013. CA. Cancer J. Clin..

[10] Desantis C.E., Ma J., Goding Sauer A., Newman L.A., Jemal A. (2017). Breast cancer statistics, 2017, racial disparity in mortality by state. CA. Cancer J. Clin..

[11] Youlden D.R., Cramb S.M., Yip C.H., Baade P.D. (2014). Incidence and mortality of female breast cancer in the Asia-pacific region. Cancer Biol. Med..

[12] Ghoncheh M., Momenimovahed Z., Salehiniya H. (2016). Epidemiology, Incidence and Mortality of Breast Cancer in Asia. Asian Pac. J. Cancer Prev..

[13] Gomez S.L., Von Behren J., McKinley M., Clarke C.A., Shariff-Marco S., Cheng I., Reynolds P., Glaser S.L. (2017). Breast cancer in Asian Americans in California, 1988−2013: Increasing incidence trends and recent data on breast cancer subtypes. Breast Cancer Res. Treat..

[14] Williams L.J., Fletcher E., Douglas A., Anderson E.D.C., McCallum A., Simpson C.R., Smith J., Moger T.A., Peltola M., Mihalicza P. (2018). Retrospective cohort study of breast cancer incidence, health service use and outcomes in Europe: A study of feasibility. Eur. J. Public Health.

[15] Abdulrahman G.O., Rahman G.A. (2012). Epidemiology of breast cancer in Europe and Africa. J. Cancer Epidemiol..

[16] Torre L.A., Bray F., Siegel R.L., Ferlay J., Lortet-Tieulent J., Jemal A. (2015). Global Cancer Statistics 2012. CA Cancer J. Clin..

[17] Clèries R., Rooney R.M., Vilardell M., Espinàs J.A., Dyba T., Borras J.M. (2018). Assessing predicted age-specific breast cancer mortality rates in 27 European countries by 2020. JM Clin. Transl. Oncol..

[18] Ghoncheh M., Mohammadian-Hafshejani A., Salehiniya H. (2015). Incidence and Mortality of Breast Cancer and their Relationship to Development in Asia. Asian Pac. J. Cancer Prev..

[19] McPherson K., Steel C.M., Dixon J.M. (2000). ABC of Breast Diseases: Breast Cancer-epidemiology, Risk Factors, and Genetics. BMJ.

[20] National Cancer Institute: Surveillance, Epidemiology, and End Results SEER Cancer Statistics Review 1975–2010. http://seer.cancer.gov/csr/1975_2010/results_single/sect_01_table12_2pgs.pdf.

[21] Trock B.J., Hilakivi-Clarke L., Clarke R. (2006). Meta-Analysis of Soy Intake and Breast Cancer Risk. JNCI.

[22] Brennan S.F., Cantwell M.M., Cardwell C.R., Velentzis L.S., Woodside J.V. (2010). Dietary patterns and Breast Cancer Risk: A Systematic Review and Meta-analysis. AJCN.

[23] Mayne S.T., Playdon M.C., Rock C.L. (2016). Diet, Nutrition, and Cancer: Past, present and Future. Nat. Rev. Clin. Oncol..

[24] Shannon J., White E., Shattuck A.L., Potter J.D. (1996). Relationship of Food Groups and Water Intake to Colon Cancer Risk. CEBP.

[25] Slattery M.L., Caan B.J., Anderson K.E., Potter J.D. (1999). Intake of Fluids, and methylxanthine-containing beverages: Association with Colon Cancer. IJC.

[26] Tang R., Wang J.Y., Lo S.K., Hsieh L.L. (1999). Physical activity, water intake and risk of colorectal cancer in Taiwan: A hospital-based case control study. Int. J. Cancer.

[27] Altieri A., Vecchia C.L., Negri E. (2003). Fluid Intake and Risk of Bladder and other cancers. Eur. J. Clin. Nutr..

[28] Bar Y., Yuan H., Li J., Tang Y., Pu C., Han P. (2014). Relationship between bladder cancer and total fluid intake: A meta-analysis of epidemiological evidence. World J. Surg. Oncol..

[29] EFSA (2010). Scientific opinion on dietary reference values for water. EFSA J..

[30] Medicine I.O. (2005). Panel on Dietary Reference Intakes for Electrolytes and Water: Dietary Reference Intakes for Water, otassium, Sodium, Chloride and Sulfate.

[31] Popkin B.M., D’Anci K.E., Rosenberg I.H. (2010). Water, hydration, and health. Nutr. Rev..

[32] Perrier E., Demazieres A., Girard N., Pross N., Osbild D., Metzger D., Guelinckx I., Klein A. (2013). Circadian variation, and responsiveness of hydration biomarkers to changes in daily water intake. Eur. J. Appl. Physiol..

[33] Lemaire J.B., Wallace J.E., Dinsmore K., Lewin A.M., Ghali W.A., Roberts D. (2010). Physician nutrition and cognition during work hours: Effect of a nutrition-based intervention. BMC Health Serv. Res..

[34] McKiernan F., Houchins J.A., Mattes R.D. (2008). Relationships between human thirst, hunger, drinking, feeding. Physiol. Behav..

[35] Malisova O., Bountziouka V., Panagiotakos D.B., Zampelas A., Kapsokefalou M. (2012). The water balance questionnaire: Design, reliability, and validity of a questionnaire to evaluate water balance in the general population. Int. J. Food Sci. Nutr..

[36] Cotter J.D., Thornton S.N., Lee J.K., Laursen P.B. (2014). Are we being drowned in hydration advice? Thirsty for more?. Extrem. Physiol. Med..

[37] Drewnowski A., Rehm C.D., Constant F. (2013). Water and beverage consumption among adults in the United States: Cross-sectional study using data from NHANES 2005−2010. BMC Public Health.

[38] Watanabe T., Nakaya N., Kurashim K., Kuriyama S., Tsubono Y., Tsuji I. (2004). Constipation, laxative use and risk of colorectal cancer. The Miyagi Cohort Study. EJC.

[39] Roberts M.C., Millikan R.C., Galanko J.A., Martin C., Sandler R.S. (2003). Constipation, laxative use, and colon cancer in a North Carolina population. Am. J. Gastroenterol..

[40] Maruti S.S., Lampe J.W., Potter J.D., Ready A., White E. (2008). A Prospective Study of Bowel Motility and Related Factors on Breast Cancer Risk. CEBP.

[41] Rose D.P., Goldman M., Connolly J.M., Strong L.E. (1991). High-fiber diet reduces serum estrogen concentrations in premenopausal women. Am. J. Clin. Nutr..

[42] Neale G. (1983). Effects of fiber on the entero-hepatic circulation of estradiol. Fiber in Human and Animal Nutrition Bulletin: Dietary Fiber Symposium.

[43] Micozzi M.S., Carter C.L., Albanes D., Taylor P.R., Licitra L.M. (1989). Bowel Function and Breast Cancer in US Women. AJPH.

[44] Mcintyre G.I. (2006). Cell Hydration as the primary Factor in Carcinogenesis. A Unifying Concept. Med. Hypotheses.

[45] Stookey J.D., Belderson P.E., Russell J.M., Barker M.E. (1996). Relationship of food groups and water intake to colon cancer risk. CEBP.

[46] Cade J.E., Burley V.J., Warm D.L., Thompson R.L., Margetts B.M. (2004). Food frequency questionnaires: A review of their design, validation and utilization. Nutr. Res. Rev..

[47] Spungen J.H., Goldsmith R., Stahl Z., Reifen R. (2013). Desalination of water. Nutritional Considerations. IMAJ.

[48] Lee H.P., Gourley L., Duffy S.W., Esteve J., Lee J., Day N.E. (1991). Dietary effects on breast-cancer risk in Singapore. Lancet.

[49] Graham S., Hellmann R., Marshall J., Freudenheim J., Vena J., Swanson M., Zielezny M., Nemoto T., Stubbe N., Raimondo T. (1991). Nutritional epidemiology of postmenopausal breast cancer in western New York. Am J. Epidemiol..

[50] Freudenheim J.L., Marshall J.R., Vena J.E., Laughlin R., Brasure J.R., Swanson M.K., Nemoto T., Graham S. (1996). Premenopausal breast cancer risk and intake of vegetables, fruits, and related nutrients. J. Natl. Cancer Inst..

[51] Farvid M.S., Chen W.Y., Michels K.B., Cho E., Willett W.C., Eliassen A.H. (2016). Fruit and vegetable consumption in adolescence and early adulthood and risk of breast cancer: Population-based cohort study. BMJ.

[52] Shield K.D., Soerjomataram I., Rehm J. (2016). Alcohol Use and Breast Cancer: A Critical Review. Alcohol. Clin. Exp. Res..

